# Peripheral Precocious Puberty Revealing McCune-Albright Syndrome in a Three-Year-Old Girl: A Case Report

**DOI:** 10.7759/cureus.108084

**Published:** 2026-05-01

**Authors:** Omayma El Athmani, Khadija Mouaddine, Bouchra Chkirate

**Affiliations:** 1 Department of Pediatric Rheumatology, Nephrology and Cardiology, Children’s Hospital, Ibn Sina University Hospital Center, Mohammed V University, Rabat, MAR

**Keywords:** aromatase inhibitor, mccune-albright syndrome, ovarian cyst, pediatric endocrinology, peripheral precocious puberty

## Abstract

McCune-Albright syndrome (MAS) is a rare genetic disorder characterized by the triad of café-au-lait skin pigmentation, fibrous dysplasia, and peripheral precocious puberty. We report the case of a three-year-old girl presenting with recurrent vaginal bleeding and progressive breast development. Clinical examination revealed café-au-lait macules, and hormonal evaluation showed elevated estradiol levels with suppressed gonadotropins.

Pelvic ultrasound demonstrated ovarian cysts. Bone age was advanced compared to chronological age. Additional imaging with technetium-99m bone scintigraphy revealed increased radiotracer uptake in multiple long bones, suggestive of increased bone turnover and supporting skeletal involvement.

These findings were consistent with a diagnosis of MAS. The patient was treated with letrozole, resulting in clinical improvement.

This case highlights the importance of considering MAS in cases of peripheral precocious puberty and emphasizes the role of multimodal clinical and imaging assessment in supporting the diagnosis.

## Introduction

McCune-Albright syndrome (MAS) is a rare disorder caused by postzygotic activating mutations of the GNAS gene, leading to mosaic involvement of multiple tissues [1]. It is classically defined by a triad of café-au-lait macules, fibrous dysplasia of bone, and endocrine hyperfunction, most commonly peripheral precocious puberty in girls [2].

Peripheral precocious puberty in MAS results from autonomous ovarian estrogen secretion, usually related to recurrent ovarian cyst formation [3]. Clinical manifestations include vaginal bleeding, breast development, and accelerated growth with advanced bone maturation [4].

Although the diagnosis is primarily clinical, imaging plays an essential role in identifying ovarian and skeletal involvement [5].

We report a case of MAS revealed by peripheral precocious puberty in a young girl, highlighting diagnostic challenges and therapeutic considerations.

## Case presentation

A three-year-old girl was admitted for recurrent vaginal bleeding over a period of three months, associated with progressive breast development.

**Figure 1:**
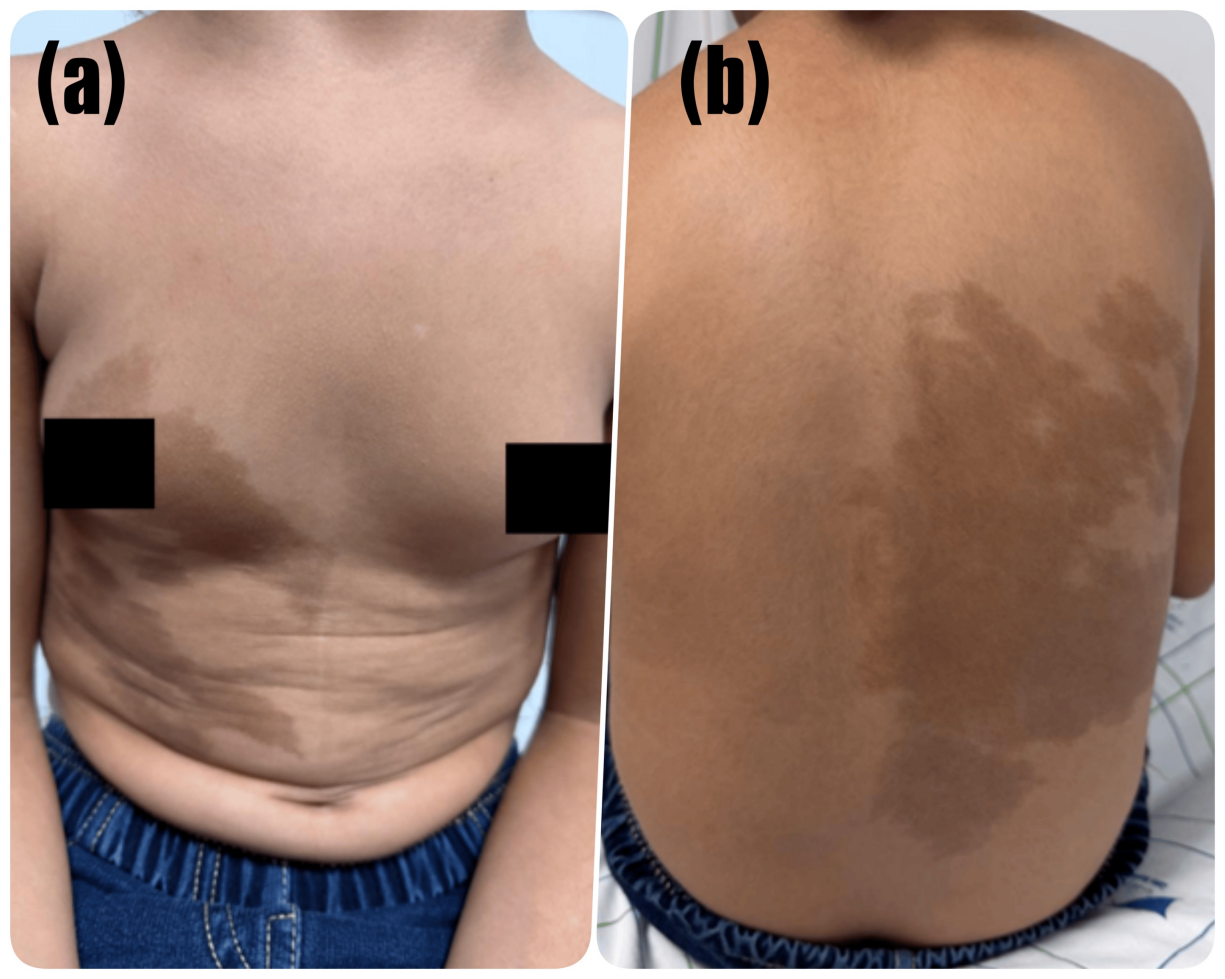
Café-au-lait macules in a child with McCune-Albright syndrome (a) Anterior view showing large café-au-lait macules over the trunk with irregular borders respecting the midline. (b) Posterior view demonstrating extensive hyperpigmented macules with irregular “coast of Maine” borders characteristic of McCune-Albright syndrome.

Physical examination revealed Tanner stage II breast development and multiple café-au-lait macules with irregular borders (Figure 1). No axillary or pubic hair was observed.

Hormonal evaluation showed elevated estradiol levels with suppressed luteinizing hormone (LH) and follicle-stimulating hormone (FSH), consistent with peripheral precocious puberty.

The main clinical and laboratory findings are summarized in Table 1.

**Table 1:** Hormonal laboratory findings Hormonal evaluation showing elevated estradiol levels with suppressed gonadotropins, consistent with gonadotropin-independent precocious puberty.

Parameter	Result	Reference range
Estradiol	179.19 pg/mL	<20 pg/mL (prepubertal girls)
Luteinizing hormone	<0.10 mIU/mL	<0.3 mIU/mL
Follicle-stimulating hormone	<0.10 mIU/mL	<4 mIU/mL

**Figure 2:**
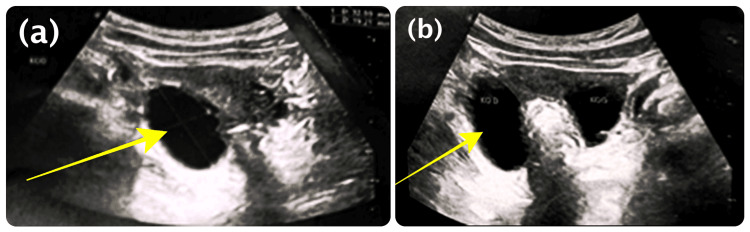
Pelvic ultrasound demonstrating ovarian cysts (a) Well-defined anechoic ovarian cyst (arrow), consistent with a functional cyst. (b) Bilateral ovarian cysts of varying sizes.

Pelvic ultrasound demonstrated a well-defined anechoic ovarian cyst (Figure 2a) and bilateral ovarian cysts of varying sizes (Figure 2b).

Bone age assessment revealed advanced skeletal maturation compared to chronological age.

**Figure 3:**
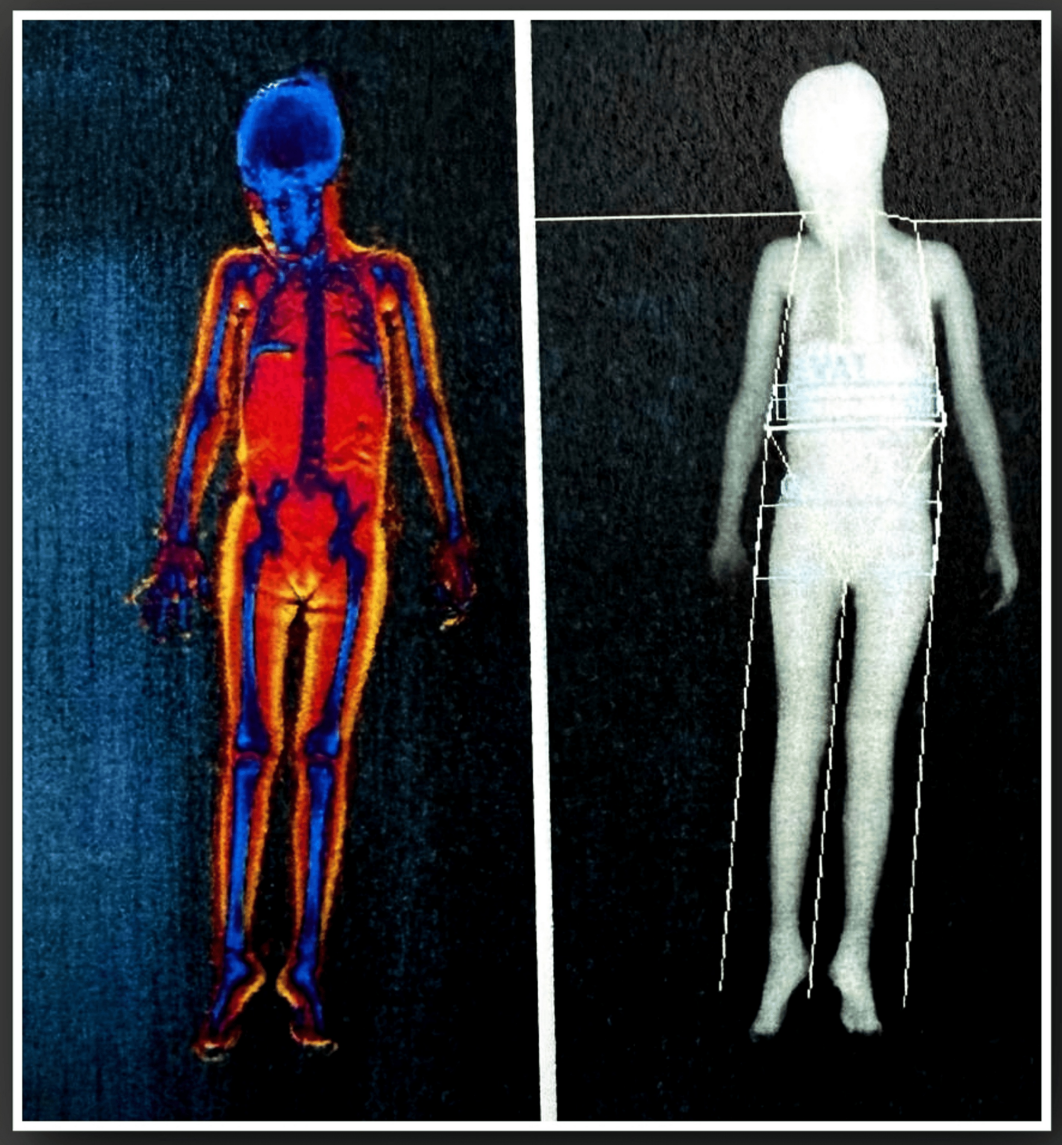
Whole-body technetium-99m bone scintigraphy The scan demonstrates increased radiotracer uptake in multiple long bones, indicating increased bone turnover and supporting skeletal involvement in the clinical context of McCune-Albright syndrome.

A technetium-99m bone scintigraphy was performed and demonstrated increased radiotracer uptake in multiple long bones, suggestive of increased bone turnover and supporting skeletal involvement (Figure 3).

Based on the association of café-au-lait macules, peripheral precocious puberty, and imaging findings, a diagnosis of MAS was considered.

The patient was started on letrozole, an aromatase inhibitor, with good clinical tolerance.

During follow-up, a reduction in the frequency of vaginal bleeding episodes was observed. No progression of breast development was noted, and growth velocity remained stable. No clinical worsening was observed, and no new endocrine abnormalities were detected. Long-term follow-up is ongoing to assess sustained clinical response and disease progression.

## Discussion

MAS is a rare disorder with variable clinical expression due to postzygotic activating mutations of the GNAS gene, resulting in mosaic involvement of affected tissues [1]. Peripheral precocious puberty is the most common endocrine manifestation in girls and is often the presenting feature [2].

The diagnosis of MAS in this case is based on a combination of clinical, hormonal, and imaging findings. The presence of café-au-lait macules associated with peripheral precocious puberty strongly suggested the diagnosis.

Pelvic ultrasound findings of ovarian cysts are characteristic and reflect autonomous estrogen secretion, which is responsible for the clinical manifestations observed in these patients [3].

Fibrous dysplasia represents the skeletal component of MAS. While initial radiographic findings may be non-specific, bone scintigraphy can provide valuable information. In our case, scintigraphy demonstrated increased radiotracer uptake in multiple long bones, supporting skeletal involvement. While bone scintigraphy is not specific, it is a sensitive imaging modality widely used to evaluate the extent of skeletal involvement in fibrous dysplasia [4]. These findings are compatible with fibrous dysplasia in the appropriate clinical context, although histological confirmation was not performed [5].

Other causes of peripheral precocious puberty should be considered in the differential diagnosis, including isolated ovarian cysts, estrogen-secreting ovarian tumors, exogenous estrogen exposure, and adrenal disorders such as congenital adrenal hyperplasia [6].

Management of MAS-related peripheral precocious puberty remains challenging and is primarily aimed at reducing estrogen production and its clinical effects. Several therapeutic options have been described, including tamoxifen, medroxyprogesterone acetate, and aromatase inhibitors [7]. Letrozole, a third-generation aromatase inhibitor, was selected in our case due to its availability, ease of administration, favorable tolerance profile, and growing evidence supporting its efficacy in reducing estrogen production and controlling symptoms.

The clinical evolution in our patient was favorable, with a reduction in vaginal bleeding episodes and stabilization of pubertal progression. No clinical worsening or new endocrine abnormalities were observed during follow-up. Long-term follow-up is ongoing and will be necessary to assess sustained treatment efficacy and disease progression.

This case highlights the importance of recognizing early signs of MAS and emphasizes the role of a multidisciplinary approach combining clinical, hormonal, and imaging findings in establishing the diagnosis and guiding management [8].

## Conclusions

MAS should be considered in young girls presenting with peripheral precocious puberty and café-au-lait macules. Imaging plays a key role in supporting the diagnosis. Early recognition allows appropriate management and helps prevent complications related to prolonged estrogen exposure. A multidisciplinary approach and regular follow-up are essential to optimize patient outcomes.
